# Inhibition of Signal Transducer and Activator of Transcription 3 (STAT3) reduces neonatal hypoxic‐ischaemic brain damage

**DOI:** 10.1111/jnc.13490

**Published:** 2016-01-11

**Authors:** Mariya Hristova, Eridan Rocha‐Ferreira, Xavier Fontana, Laura Thei, Rheanan Buckle, Melina Christou, Supanida Hompoonsup, Naomi Gostelow, Gennadij Raivich, Donald Peebles

**Affiliations:** ^1^ UCL Institute for Women's HealthMaternal & Fetal MedicinePerinatal Brain Repair GroupLondon WC1E 6HXUK; ^2^ Cell Growth and Regeneration LabMRC Laboratory for Molecular Cell BiologyUniversity College LondonWC1E 6BTUK

**Keywords:** astroglia, microglia, neonatal hypoxia‐ischaemia, neuroprotection, STAT3

## Abstract

Hypoxic‐ischaemic encephalopathy is a leading cause of child death, with high mortality and morbidity, including cerebral palsy, epilepsy and cognitive disabilities. Hypoxia‐ischaemia (HI) strongly up‐regulates Signal Transducer and Activator of Transcription 3 (STAT3) in the immature brain. Our aim was to establish whether STAT3 up‐regulation is associated with neonatal HI‐brain damage and evaluate the phosphorylated STAT3‐contribution from different cell types in eliciting damage. We subjected postnatal day seven mice to unilateral carotid artery ligation followed by 60 min hypoxia. Neuronal STAT3‐deletion reduced cell death, tissue loss, microglial and astroglial activation in all brain regions. Astroglia‐specific STAT3‐deletion also reduced cell death, tissue loss and microglial activation, although not as strongly as the deletion in neurons. Systemic pre‐insult STAT3‐blockade at tyrosine 705 (Y705) with JAK2‐inhibitor WP1066 reduced microglial and astroglial activation to a more moderate degree, but in a pattern similar to the one produced by the cell‐specific deletions. Our results suggest that STAT3 is a crucial factor in neonatal HI‐brain damage and its removal in neurons or astrocytes, and, to some extent, inhibition of its phosphorylation via JAK2‐blockade reduces inflammation and tissue loss. Overall, the protective effects of STAT3 inactivation make it a possible target for a therapeutic strategy in neonatal HI.

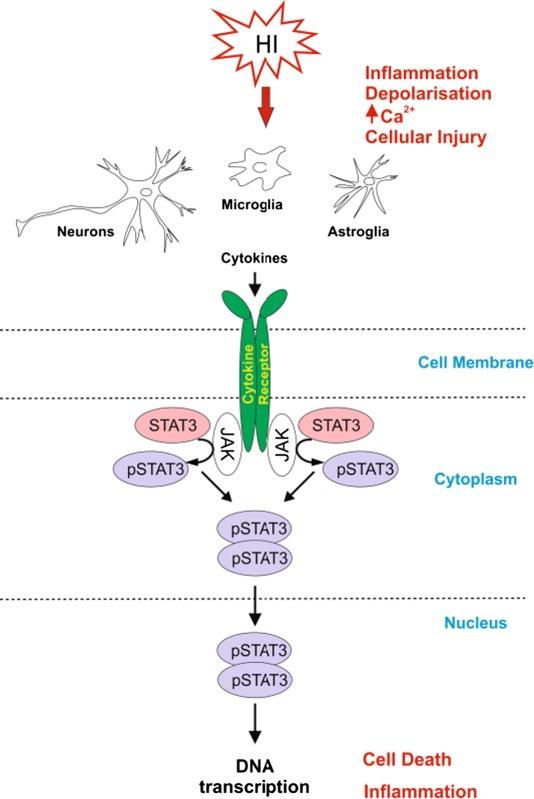

Current data show that neuronal and astroglial STAT3 molecules are involved in the pathways underlying cell death, tissue loss and gliosis following neonatal hypoxia‐ischaemia, but differ with respect to the target of their effect. Y705‐phosphorylation contributes to hypoxic‐ischaemic histopathology. Protective effects of STAT3 inactivation make it a possible target for a therapeutic strategy in neonatal hypoxia‐ischaemia.

**Abbreviations used**
BW: body weight
DMSO: dimethyl sulfoxide
GFAP: glial fibrillary acidic protein
HIE: hypoxic ischaemic encephalopathy
HI: hypoxia ischaemia
i.p.: intraperitoneal
JAK: Janus Kinase
NE: neonatal encephalopathy
OLV: optical luminosity values
PBS: phosphate buffered saline
pSTAT3: phosphorylated Signal Transducer and Activator of Transcription 3
SD: standard deviation
SEM: standard error of the mean
STAT3: Signal Transducer and Activator of Transcription 3
SV: subventricular
TH: therapeutic hypothermia
TUNEL: terminal deoxynucleotidyl transferase dUTP nick end labelling

Neonatal hypoxic ischaemic encephalopathy affects 1–3 per 1000 live term births in high‐income countries, with rates 5–10 times higher in low‐income settings. About 40% of affected infants die in the neonatal period and an additional 30% have lifelong neurological deficits, including cerebral palsy, epilepsy and cognitive disabilities causing substantial socio‐economic burden to the individual, family and healthcare system (Kelen and Robertson 2010).

Signal Transducer and Activator of Transcription 3 (STAT3), a member of the STAT family, is strongly up‐regulated by hypoxia ischaemia (HI) in the immature brain (Shrivastava *et al*. 2013). STAT3 activation is due to JAK2‐dependent phosphorylation of Tyr‐705 (Y705), and JAK2‐independent phosphorylation of Ser‐727 residues (Aggarwal *et al*. 2009). Phosphorylation of both sites is associated with post‐ischaemic events (Yang *et al*. 2005; Heusch *et al*. 2011) and phosphorylated STAT3 (pSTAT3) co‐localizes with cell death‐associated DNA degradation (Wen *et al*. 2001). JAK2‐dependent pharmacological inhibition of pSTAT3 reduces occlusion/reperfusion‐induced damage in adult models of middle cerebral artery occlusion (Lei *et al*. 2011). The neuroprotective effects of estradiol (Dziennis *et al*. 2007) and IL6 (Jung *et al*. 2011) depend on inhibition of pSTAT3. Therapeutic hypothermia also reduces pSTAT3 levels (Choi *et al*. 2011).

In addition to neurons, in adult animal models of ischaemia, activated STAT3 is also present in blood vessel endothelia, astrocytes and microglia (Planas *et al*. 1996; Justicia *et al*. 2000; Choi *et al*. 2011), potentially associating it with neuroinflammation and the associated tissue repair (Yi *et al*. 2007). Although some studies have looked into the activation and up‐regulation of STAT3 following neonatal HI relating it to the balance between pro‐ and anti‐inflammatory cytokines (Shrivastava *et al*. 2013), the overall function and precise role of this transcription factor in different cell types of the neonatal injured brain remains unclear. Using global inhibition of STAT3 phosphorylation and brain cell type‐specific deletion of the STAT3 gene, the study reveals a critical role of neuronal and astroglial STAT3 in promoting neuronal damage and glial activation in response to neonatal HI‐insult.

## Materials and methods

### Animal strains and breeding strategy

The animals used in the WP1066 inhibitor experiments were from the C57/Bl6 strain obtained from Charles River (Kent, UK). Mice carrying floxed STAT3 allele (*STAT3* ^*F/F*^) on SVJ129 background (Takeda *et al*. 1998) were kindly provided by Prof. Shizuo Akira from the Institute for Molecular and Cellular Biology, Osaka University, and were initially maintained as heterozygotes (*STAT3* ^*F/wt*^) by crossing them with C57/Bl6 obtained from Charles River (Kent, UK). Animals expressing cre recombinase driven by synapsin promoter (syn::Cre, C57/Bl6 strain) were provided by Dr Axel Behrens from the Mammalian Genetics Laboratory, Cancer Research UK, and animals expressing cre recombinase under the control of glial fibrillary acidic protein promoter (GFAP::Cre, FVB strain) were from Jackson Labs (USA, http://jaxmice.jax.org/strain/004600.html, Charles River, Kent, UK). The wild‐type littermate controls in the cell type‐specific STAT3 deletion experiments were thus on mixed C57/Bl6 and SVJ129 background for the neuron‐specific STAT3 deletion or on mixed C57/Bl6 and FVB background for the astroglia‐specific deletion.

For cell type‐specific STAT3 deletion studies, Syn::Cre animals were crossed twice with *STAT3* ^*F/F*^ mice (Zhu *et al*. 2001; Ruff *et al*. 2012), to obtain homozygous CNS neuron‐specific deletion of STAT3 (*STAT3* ^*∆S*^). To prevent germline STAT3 deletion due to testicular expression of the synapsin promoter (Hoesche *et al*. 1993; Street *et al*. 2005) only female cre‐positive (*STAT3* ^*∆S*^) and male cre‐negative *STAT3* ^*F/F*^ mice were used for breeding to generate *STAT3* ^*∆S*^ mutants and *STAT3* ^*F/F*^ littermate controls with Mendelian distribution. A similar approach was used for the generation of animals with homozygous astroglia‐specific deletion of STAT3 (*STAT3* ^*∆G*^), by crossing GFAP::Cre mice twice with *STAT3* ^*F/F*^ animals.

### DNA isolation and genotyping

DNA extraction was performed with the ‘Wizard’ Genomic DNA purification system according to manufacturer's instructions (Promega, Southampton, UK), using tail tips taken before the perfusion. Specific oligonucleotide primers (Invitrogen, Paisley, UK) were used for genotyping.

STAT3 flox forward primer: 5′‐CCTGAAGACCAAGTTCATCTGTGTGAC‐3′

STAT3 flox reverse primer: 5′‐CACACAAGCCATCAAACTCTGGTCTCC‐3′

GFAP‐cre forward primer: 5′‐ACTCCTTCATAAAGCCCT‐3′

GFAP‐cre reverse primer: 5′‐ATCACTCGTTGCATCGACCG‐3′

Synapsin‐cre forward: 5′‐AGCTTCAGCACCGCGGACAGT‐3′

Synapsin‐cre reverse: 5′‐TCGTTGCATCGACCGGTAATG‐3′

### HI Insult

All animal experiments and care protocols were carried out according to the UK Animals (Scientific Procedures) Act 1986 and approved by the Home Office. The ARRIVE guidelines were followed. All experiments involved postnatal day 7 mice (P7) bred in house.

The surgical procedures were performed as previously described (Kendall *et al*. 2006). Briefly, male and female postnatal day 7 (P7) mice were anaesthetised with isofluorane (5% induction, 1.5% maintenance). The left common carotid artery was permanently occluded with 8/0 polypropylene suture and the wound closed with tissue glue. The mice were left to recover at 36°C and returned to the dam for 2 h. The pups were then placed in a hypoxia chamber and exposed to humidified 8% oxygen/92% nitrogen ( 3 L/min) at 36°C for 60 min.

In the rodent brain at P7, some cell types are at a developmental stage similar to a mid‐third‐trimester human fetus or new‐born infant, with complete cortical neuronal layering, involuted germinal matrix, and slightly myelinated white matter (Vannucci and Vannucci 1997).The rodent HI model at P7, though slightly preterm, presents phenotypical similarities to the grey and white matter injury observed in humans, i.e. tissue loss, cell‐death‐mediated apoptosis, microglia‐mediated immune response and astrogliosis as well as alteration in neurobehavioural performance (Vannucci and Vannucci 1997).

### Pharmacological treatment

The JAK2 inhibitor WP1066 (Calbiochem, Watford, UK) was dissolved in 100% dimethyl sulfoxide (DMSO) and administered intraperitoneally at two doses of 40 mg/kg body weight (BW) (Iwamaru *et al*. 2007; Horiguchi *et al*. 2010) 20 min prior and then again directly after HI, as the mean half‐life of WP1066 is 4.5 h (Madden *et al*. 2006). The WP1066‐treated animals received two injections of 0.25 μL/g BW. The control groups received a corresponding amount of DMSO. The animals were left to survive for 48 h and the brains were collected for histological analysis.

### Western blot analysis

For western blot analysis, the animals were sacrificed at 1 h following HI. Western blot analysis was performed as previously described (Nateri *et al*. 2005). For immunoblotting, we used antibody against pSTAT3 (Y705) (New England Biolabs, Hitchin, UK).

### Tissue sample preparation

For histological assessment, the animals were sacrificed at 48 h post‐HI by intraperitoneal injection of pentobarbitone and perfused with 30 mL 4% paraformaldehyde in phosphate buffered saline (PBS). The brains were then removed, post‐fixed in 4% paraformaldehyde in PBS for 1 h at 4°C, and cryoprotected for 24 h in phosphate‐buffered 30% sucrose solution as described before (Möller *et al*. 1996). The brains were then frozen on dry ice, cut on a cryostat into sequential 40‐μm sections and stored at −80°C until required.

### Immunohistochemistry and histological analysis

**Figure 1:**
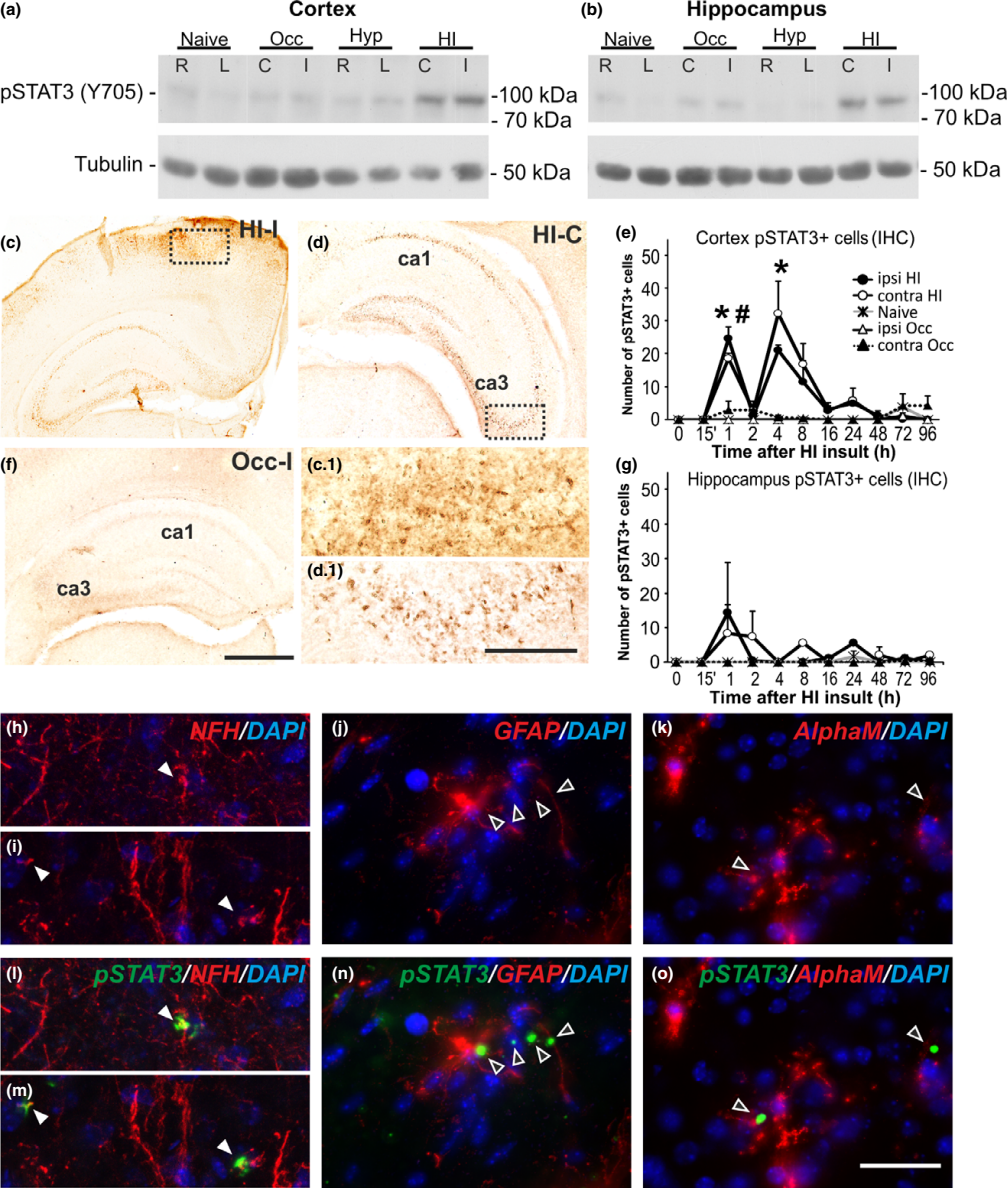
**pSTAT3 increase in cerebral cortex and hippocampus following HI‐insult in P7 mice. (a and b)** Western Blots for pSTAT3 (Y705) in cerebral cortex (a) and hippocampus (b) from naïve C57Bl/6 animals, animals with unilateral carotid occlusion/ischemia (Occ) or hypoxia (Hyp) – 60 min exposure to 8% oxygen and 1 h recovery; and animals with unilateral occlusion followed by 60 min hypoxia (HI) and 1 h recovery. Tubulin protein levels served as controls. L and R – left and right hemispheres in naïve and hypoxic animals, (c) and (i) – contralateral and ipsilateral in animals with unilateral occlusion only, and in those with HI‐insult. The strong and bilateral pSTAT3 increase in cortex and hippocampus is only present following HI‐insult (a and b). No STAT3 activation is observed in cortex or hippocampus of naïve or animals subjected to occlusion (Occ) or hypoxia alone (Hyp). **(c, d and f)** HI‐insult strongly increased ipsilateral (c) and contralateral (d) pSTAT3 immunoreactivity in cortex and hippocampus (rostral part of parietal isocortex) at 1 h post‐HI. Occlusion alone (Occ) produces no change in pSTAT3 immunoreactivity (f). (c.1 and d.1) Magnified images of the dotted boxes in cerebral cortex (c) and the CA3 region of hippocampus (d), respectively. **(e and g)** Number of pSTAT3 (Y705)+ cells in ipsilateral and contralateral cerebral cortex (e) and hippocampus (g) 0–96 h post‐HI (Mean ± SEM,* n *=* *4 animals per group). Note the bilateral peak at 1 and 4 h in cortex (e) and at 1‐2 h in hippocampus (g) in the animals with unilateral occlusion followed by 60 min HI compared to unilateral occlusion alone (Occ) or naïve animals (*t*‐test, ipsilateral cortex *p = 0.0063 for 1 h, *p* = 0.046 for 4 h; contralateral cortex #*p* = 0.0015 for 1 h). **(h–o)** Immunofluorescence for chick polyclonal anti‐neurofilament‐H (NFH, h, i, l, m), rat monoclonal anti‐glial fibrillary acidic protein (GFAP, j and n) and rat monoclonal anti‐αM integrin subunit (k and o) in cerebral cortex at 1 h following neonatal HI, in red, superimposed on the rabbit polyclonal anti‐pSTAT3 (Y705) fluorescence in green and nuclear DAPI fluorescence in blue. Note the co‐localization of pSTAT3 and NFH (l and m, full arrows) and the lack of such co‐localization with GFAP+ astroglia (n, empty arrows) and αM+ microglial cells (o, empty arrows), suggesting neuronal expression of pSTAT3 at this time point post‐HI. *Scale bars*: c, d, f = 400 μm; c.1, d.1, h–o = 50 μm.

Five cryosections from each brain (400 μm apart), obtained at 48 h post‐HI, were rehydrated in distilled water and stained using immunohistochemistry as previously described (Möller *et al*. 1996). Briefly, the sections were incubated overnight with primary antibodies summarized in Table 1, for 2 h with biotinylated goat anti‐rabbit, ‐rat or ‐hamster (1 : 100, Vector, Peterborough, UK) secondary antibody, followed by incubation with Avidin‐Biotinylated horseradish peroxidase Complex (Vector) and visualization with diaminobenzidine/H_2_O_2_ (Fisher Scientific, Loughborough, UK). αX immunoreactivity was enhanced with Co/Ni. Overall, GFAP immunoreactivity was detected with the rabbit polyclonal anti‐GFAP antibody. However, for the double immunofluorescence with pSTAT3 in Fig. 1j and n we used rat monoclonal anti‐GFAP antibody, to avoid cross‐staining with the rabbit polyclonal antibody against pSTAT3 (see Table 1).

**Table 1:** List of antibodies used for immunohistochemistry

Antibody against	Concentration	Host species	Secondary antibody	Obtained from
αM integrin subunit	1 : 5000	Rat	Goat anti‐rat	Serotec, Kidlington, UK Cat Nr MCA711
αX integrin subunit	1 : 400	Hamster	Goat anti‐hamster	Pierce, Paisley, UK Cat Nr MA11C5
GFAP	1 : 6000	Rabbit polyclonal	Goat anti‐rabbit	Dako, Ely, UK Cat Nr Z0334
GFAP	1 : 6000	Rat monoclonal	Goat anti‐rabbit	Zymed, Loughborough, UK Cat Nr 13‐0300
pSTAT3 (Y705)	1 : 400	Rabbit	Goat anti‐rabbit	New England Biolabs, Hitchin, Cat Nr 9131
Neurofilament‐H	1 : 10000	Chicken	Goat anti‐chicken	Abcam, Cambridge, UK Cat Nr ab4680

Five further cryosections from each brain with the same spacing were stained using Terminal transferase mediated d‐UTP nick end labelling (TUNEL) (Roche, Burgess Hill, UK). The staining procedure followed the manufacturer protocol with Co/Ni enhancement.

Five more sections per brain with the same spacing were stained with cresyl violet (Nissl).

For the double immunofluorescence with pSTAT3 in Fig. 1h, i, l and m, we used Neurofilament‐H (h, i, l and m) as a major component of the neuronal cytoskeleton and thus a neuronal marker, GFAP (j and n) and αM (k and o), and followed the above‐described protocol replacing the biotinylated secondary antibodies with fluorescently labelled ones.

### Infarct volume measurement

The cresyl violet‐stained sections were scanned and imported into Optimas 6.5 image analysis software (Bothell, WA, USA). The areas of intact staining in cortex, pyriform cortex, hippocampus, striatum, thalamus and external capsule were outlined and measured bilaterally. The percentage tissue loss was then calculated by converting the measured injured and uninjured areas into square millimetres and then converting the volume by multiplying by 400 μm. The sum of these volumes was then used to calculate the percentage of surviving brain tissue as injured/uninjured volume × 100 (Kendall *et al*. 2006).

### pSTAT3 immunoreactivity

To analyse the pSTAT3 immunoreactivity following HI, we counted the number of positive cells bilaterally in three different optical fields at × 40 magnification in cortex and hippocampus at 0, 15 min, 1, 2, 4, 8, 16, 24, 48, 72, and 96 h following HI (*n* = 4 animals per group). The counts were then averaged per animal and per group.

### AlphaM score

Immunohistochemistry for αM integrin as a marker of early microglial activation (Kloss *et al*. 1999; Hristova *et al*. 2010; Kendall *et al*. 2011a; Lange *et al*. 2014), was performed as previously described (Ohno *et al*. 1995; Kendall *et al*. 2006). Semi‐quantitative scores were allocated to each brain region (cortex, pyriform cortex, hippocampus, striatum, thalamus and external capsule) by an observer blinded to the treatment of the groups (Table 2).

**Table 2:** Semi‐quantitative scores for αM integrin

Score	Microglial appearance
0	No activation
1	Focal microglial activation
2	Mild phagocytic activation affecting < 50% of the region
3	Phagocytic activation affecting > 50% of the region
4	Total phagocytic activation

### AlphaX and TUNEL

Late microglial activation and phagocytosis was assessed through immunohistochemistry for αX integrin (Kloss *et al*. 1999; Hristova *et al*. 2010). To analyse the αX integrin and TUNEL immunoreactivity at 48 h following HI, the number of positive cells for each of the stains was counted bilaterally in three different optical fields at ×20 magnification in cortex, pyriform cortex, hippocampus, striatum, thalamus and external capsule. The counts were then averaged per animal and per group.

### Optical luminosity

GFAP is expressed not just by the bodies, but is also registered in the processes of astroglial cells. In order to quantify the total intensity of the GFAP staining in the tissue, we used optical luminosity values as a well‐established technique (Möller *et al*. 1996). Images for both ipsilateral and contralateral sides were captured with a Sony AVT‐Horn 3CCD colour video camera (24bit RGB, 760 × 570 pixel resolution) in three different optical fields in cortex, pyriform cortex, hippocampus, striatum, thalamus and external capsule, as well as the surrounding glass at ×20 magnification. We used Optimas 6.5 software to obtain the mean and standard deviation (SD) for optical luminosity values (OLV). SD was subtracted from the mean for each image and the resulting value was subtracted from the values obtained for the surrounding glass. (Möller *et al*. 1996).

### Statistics

Statistical significance was assessed through repeated testing using Mixed Linear Model with SPSS 13.0 software (IBM Corporation, Armonk, New York, USA), treating region as the repeated measure. For each outcome, several regions of the brain were examined. With repeated measures such as these, it is likely that the observations from a single subject will be correlated, therefore the first stage of the analysis included the observations from all the regions tested in a single mixed model with a random subject effect, to produce an estimate of the treatment effect and associated inference that accounts for the correlations in the data arising from the repeated measures. Further *post hoc* Student *t*‐tests were carried out to assess evidence for subregional differences, *p* < 0.05. For each outcome, the overall effect from the linear mixed model is reported, followed by the results from the individual regional *t*‐tests. All data are presented as Mean + SEM.

## Results

### STAT3 phosphorylation following HI‐ insult in the neonatal brain

The protein levels of pSTAT3 (Y705) were assessed through western blot analysis. Compared to littermate controls (naïve, after unilateral carotid occlusion or after hypoxia alone), postnatal day 7 (P7) mouse pups (C57/Bl6) demonstrated bilateral pSTAT3 (Y705) up‐regulation in cortex and hippocampus, at 1 h following a 60 min HI‐insult (Fig. 1a and b). In both regions and on both sides, the total protein STAT3 levels in animals exposed to combined HI‐injury remained unchanged when compared to naïve littermate controls (data not shown).

Similar HI‐mediated pSTAT3 (Y705) increase was also observed through immunohistochemistry. Figures 1c and d reveal a bilateral increase in pSTAT3 (Y705) immunoreactivity observed on the occluded (Fig. 1c and c.1) and non‐occluded (Fig. 1d and d.1) side, in the cortex (Figs 1c and c.1) and hippocampus (Figs 1c,d and d.1) at 1 h following 60 min HI‐insult. Carotid artery ligation alone did not result in detectable pSTAT3 immunoreactivity (Fig. 1f).

In terms of pSTAT3 + cell density, this bilateral up‐regulation was detected in both cortex and hippocampus as early as 1 h (Fig. 1e and g) and was still detectable at lower levels up to 24 h following HI‐insult (*n* = 4 animals per time point). Cortical pSTAT3 +  counts (Fig. 1e) revealed a biphasic, bilateral increase with peaks at 1 and 4 h, and almost no pSTAT3 +  cells at 2 h post‐HI. The hippocampus (Fig. 1g) demonstrated a single‐bilateral peak at 1 h with lower variable levels in both hemispheres at later time points. To determine the identity of the pSTAT3 +  cells at 1 h following neonatal HI insult, pSTAT3 immunoreactivity was combined with fluorescent immunostaining for neurofilament‐H (NFH), GFAP or αM integrin subunit, and counter‐stained for nuclear 4′,6‐diamidino‐2‐phenylindole (DAPI) fluorescence in dorsoparietal cortex. As shown in Figures 1h and i, and l and m there was clear co‐localization with neuronal NFH (red), but not with astroglial GFAP (Fig. 1j and n) or with microglial αM (Fig. 1k and o). We did not observe microglial and astroglial co‐localization at 1h post‐HI, but such co‐localization has been demonstrated at later time‐points, i.e. 3–72 h, by other groups (Shrivastava *et al*. 2013; D'Angelo *et al*. 2015).

### Neuronal deletion of pSTAT3 reduces brain damage following HI‐insult in the neonate

To explore the functional role of HI‐induced STAT3 activation, animals expressing one gene copy of cre recombinase under the control of the synapsin promoter were crossed twice with mice carrying 2 copies of floxed STAT3 gene (*STAT3* ^*F/F*^ *)*, to obtain animals with homozygous CNS neuron‐specific deletion of STAT3 (*STAT3* ^*∆S*^) and *STAT3* ^*F/F*^ littermate controls, on a mixed C57/Bl6xSVJ129 background.

**Figure 2:**
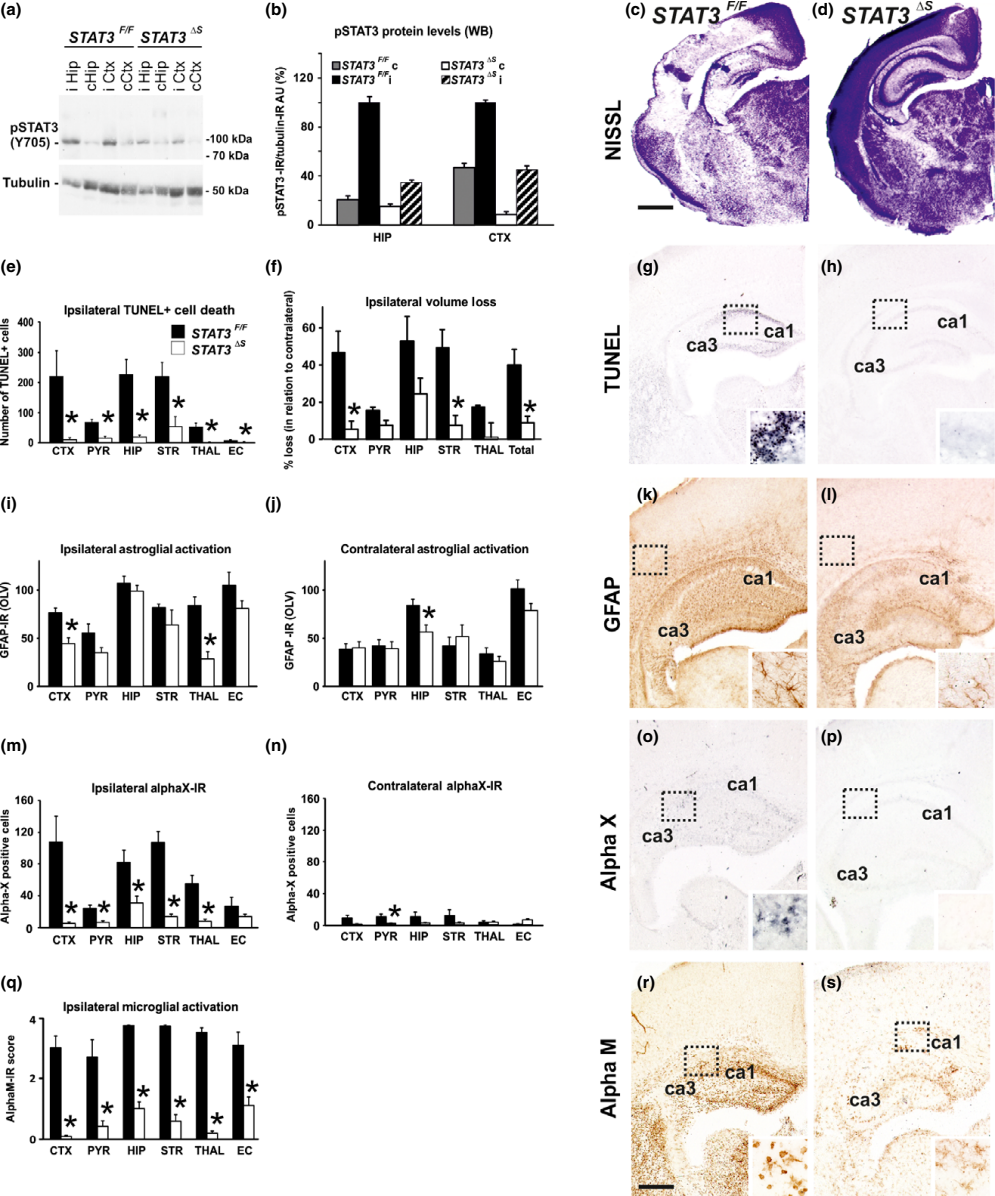
**Synapsin promoter‐specific deletion of STAT3 causes a decrease in pSTAT3 (Y705) protein levels in cortex and hippocampus, and reduces brain tissue loss, TUNEL+ cell death, reactive astrogliosis and microglial activation, following neonatal HI. (a and b)** Western Blot (a) and densitometric quantification (b) of pSTAT3 (Y705) immunoreactivity in cerebral cortex (Ctx) and hippocampus (Hip) on ipsilateral (i) and non‐occluded contralateral (c) side, 1 h after 60 min HI‐insult, with tubulin control. The ratio between pSTAT3 and tubulin immunoreactivity is shown in arbitrary units (AU) set as 100% for ipsilateral hippocampus and cortex of *STAT3* ^*F/F*^ control animals. Neuronal STAT3‐deletion (*STAT3* ^*ΔS*^) produced a clear, bilateral reduction in pSTAT3 levels (Mean ± SEM;* n *=* *5 animals per group). **(c, d and f)** Ipsilateral forebrain Nissl staining (Cresyl‐Violet, at rostral parietal level) of *STAT3* ^*F/F*^ (c) and *STAT3* ^*ΔS*^ (d) animals and quantification of ipsilateral brain tissue volume loss (f) at 48 h following HI‐insult. Neuron‐specific deletion of STAT3 (*STAT3* ^*ΔS*^, *n *=* *6) reduced volume loss (Mean ± SEM) compared to *STAT3* ^*F/F*^ littermates (*n* = 5), with significant, individual decrease (*t*‐test) in cortex (*p *=* *0.020), striatum (*p *=* *0.008), and total volume loss (*p* = 0.017). Mixed linear model, treating region as a repeated measure revealed *p* = 0.024. **(e, g and h)** TUNEL+ staining of dying brain cells with fragmented DNA at 48 h following HI‐insult – Quantification (e) (number of TUNEL+ cells per 20× eye‐field, Mean ± SEM) and histochemical overview of the ipsilateral forebrain in *STAT3* ^*F/F*^ (g) and *STAT3* ^*ΔS*^ (h) animals. Note the typical pyknotic nuclear morphology of the TUNEL+ cells observed in the *STAT3* ^*F/F*^ group (g‐ insert, hippocampus) and the lack of such cells in the *STAT3* ^*ΔS*^ group (h). Neuron‐specific deletion of STAT3 reduced TUNEL+ cell death across all 6 examined forebrain regions, with significant, individual decrease (*t*‐test) in cortex (*p *= 0.02), pyriform cortex (*p* = 0.002), hippocampus (*p* = 0.001), striatum (*p* = 0.02), thalamus *p* = (0.001), external capsule (*p* = 0.04). Mixed Linear Model, treating region as a repeated measure revealed *p* = 0.0001. **(i–l)** GFAP immunoreactivity at 48 h – Quantification of the ipsilateral (i) and contralateral, non‐occluded side (j) in optical luminosity values (OLV, Mean ± SEM), and low magnification ipsilateral overview in *STAT3* ^*F/F*^ (k) and *STAT3* ^*ΔS*^ (l) animals. The inserts in (k) and (l) show higher magnifications of the dotted regions in rostro‐parietal isocortex. Note the reduced levels of GFAP immunoreactivity in the *STAT3* ^*ΔS*^ animals, with significant, individual decrease (*t*‐test) in ipsilateral cortex (*p *=* *0.002) and thalamus (*p *=* *0.001) in (i), and in contralateral hippocampus (*p* = 0.018) in (j). Mixed Linear Model, treating region as a repeated measure revealed *p* = 0.001 for the ipsilateral, and *p* = 0.049 for the contralateral side. **(m–p)** αX+ microglia at 48 h – Numbers of αX+ cells per 20× eye‐field (Mean ± SEM) on the ipsilateral (m) and contralateral, non‐occluded side (n), and their ipsilateral distribution in *STAT3* ^*F/F*^ (o) and *STAT3* ^*ΔS*^ (p) animals. Note the intense staining and the round, phagocytic morphology of the αX+ cells in the *STAT3* ^*F/F*^ animals (o‐insert, hippocampus). Neuron‐specific STAT3 deletion significantly reduced the number of αX+cells with individual decrease (*t*‐test) in ipsilateral isocortex (*p *=* *0.006), pyriform cortex (*p *=* *0.002), hippocampus (*p *=* *0.010), striatum (*p *=* *5 × 10^−5^) and thalamus (*p *=* *0.0009) in (m); and in contralateral pyriform cortex (*p *=* *0.010) in (n). Mixed Linear Model, treating region as a repeated measure revealed *p* = 0.0001 for the ipsilateral and *p* = 0.013 for the contralateral side. **(q–s)** Activation of αM+ microglia – Ipsilateral αM microglial activation score (q, Mean ± SEM) and low magnification ipsilateral overview in *STAT3* ^*F/F*^ (r) and *STAT3* ^*ΔS*^ (s) animals. Note the strong microglial activation in *STAT3* ^*F/F*^ animals with αM+ cells showing phagocytic morphology at high magnification (r‐insert, hippocampus), compared to the *STAT3* ^*ΔS*^ brains exhibiting a ramified phenotype (s‐insert). Neuron‐specific deletion of STAT3 reduced αM+ microglial activation across all 6 examined forebrain regions, with significant, individual decrease (*t*‐test) in isocortex (*p* = 1 × 10^−5^), pyriform cortex (*p* = 0.002), hippocampus (*p* = 9 × 10^−7^), striatum (*p* = 1 × 10^−7^), thalamus (*p* = 7 × 10^−9^), external capsule (*p *=* *0.003). Mixed Linear Model, treating region as a repeated measure *p* = 0.037. *STAT3* ^*F/F*^ (*n *=* *5) and *STAT3* ^*ΔS*^ (*n *=* *6) in all assessments. CTX – cerebral iscocortex, PYR – pyriform cortex, HIP – hippocampus, STR – striatum, Thal – thalamus, EC – external capsule. *p<0.05 *Scale bars*: g, h, k, l, o, p, r and s = 600 μm; inserts=30 μm; c and d = 1200 μm.

As shown in Fig. 2a and b neuronal deletion of both copies of the floxed STAT3 gene (*STAT3* ^*∆S*^) strongly reduced western blot levels of pSTAT3 (Y705) in hippocampus and cortex compared to *STAT3* ^*F/F*^ littermate controls at 1 h following a 60 min HI‐insult (Fig. 2a and b). However, unlike the C57/Bl6 wild‐type mice (Fig. 1a and b), the relative pSTAT3 levels on the non‐occluded, contralateral side of the forebrain were lower, both in *STAT3* ^*∆S*^ and in *STAT3* ^*F/F*^ littermates.

To determine the biological impact of neuron‐specific STAT3 deletion, we next examined the effects on TUNEL+ cell death and brain volume loss, on reactive astrogliosis and on different markers or microglial activation at 48 h following a 60 min HI‐insult. Figures 2e, g and h show that Syn::cre‐mediated deletion of STAT3 (*STAT3* ^*∆S*^) markedly reduced the number of TUNEL+ cells compared to *STAT3* ^*F/F*^ littermate controls (Fig. 2e, g and h). The TUNEL+ cells displayed the typical pyknotic nuclear morphology (Fig. 2g‐insert, ipsilateral hippocampus *STAT3* ^*F/F*^). The Syn::cre‐mediated reduction was observed across all six examined forebrain regions (Mixed Linear Model, treating region as a repeated measure, *p *=* *0.0001): cerebral isocortex, pyriform cortex, hippocampus, striatum, thalamus and external capsule. Individually, all six regions revealed a significant, 70–90% decrease in the number of TUNEL+ cells in *STAT3* ^*∆S*^ animals compared to their *STAT3* ^*F/F*^ littermates (*p* < 0.05 in *t*‐test).

A similar effect of the Syn::cre‐mediated deletion of STAT3 was also observed for forebrain tissue loss, i.e. the reduction of the volume on the ipsilateral versus the non‐occluded contralateral side. Figure 2c shows large infarct in mid‐lateral isocortex (09:00‐11:00 segment) and hippocampus of the *STAT3* ^*F/F*^ animal (Fig. 2c) and its sparing in the *STAT3* ^*∆S*^ littermate (Fig. 2d). Quantification of ipsilateral volume loss (Fig. 2f) revealed strong decrease across the different forebrain regions (Mixed Linear Model, treating region as a repeated measure, *p* = 0.024), with individual significant decrease of 80–90% in isocortex, striatum and total forebrain area (*p* < 0.05 in *t*‐test).

In addition to cell death and brain tissue loss, Syn::cre‐mediated deletion of STAT3 also decreased HI‐induced and predominantly ipsilateral reactive astrogliosis and microglial activation. Compared to *STAT3* ^*F/F*^ animals, their *STAT3* ^*∆S*^ littermates revealed less overall polyclonal GFAP‐immunoreactivity (Fig. 2i and j), with substantially reduced and more spotty areas of GFAP+ astroglial processes (Fig. 2k and l). Assessment across the different forebrain regions through Mixed Linear Model, treating region as a repeated measure revealed a clear decrease on the ipsilateral, and milder reduction on the contralateral side (*p* = 0.001 and *p* = 0.049, respectively), with individual significant decrease of 40–65% in ipsilateral isocortex and thalamus, and of 32% in contralateral hippocampus (*p* < 0.05 in *t*‐test).

While the microglial cells in the control *STAT3* ^*F/F*^ group 48 h following a 60 min HI‐insult revealed the round, phagocytic phenotype shown in the inserts of Fig. 2o and 2r, the microglial cells detected in the *STAT3* ^*∆S*^ group mainly displayed resting to activated, but still ramified morphology (Fig. 2s‐insert). Figures 2m–p show that Syn::cre‐mediated deletion of STAT3 substantially decreased microglial immunoreactivity for αX integrin subunit, a marker of phagocytic/late microglial activation (Kloss *et al*. 1999). Quantification of αX+ cells across the forebrain regions through the Mixed Linear Model,treating region as a repeated measure revealed significant ipsilateral reduction (*p* = 0.0001), with individual decreases of 50–90% in all brain regions apart from external capsule (*p* < 0.05 in *t*‐test). Although the non‐occluded, contralateral side showed much fewer αX+ cells, Syn::cre‐mediated deletion of STAT3 also led to significant contralateral reduction of the number of αX+ microglia (Mixed Linear Model treating region as a repeated measure, *p* = 0.013), with individual decrease of 90% in the contralateral pyriform cortex (*p* < 0.05 in *t*‐test).

A similar effect was also observed for microglial activation score (Fig. 2q) based on the αM integrin immunoreactivity (Fig. 2r&s). Regional assessment shown in Fig. 2q revealed a reduction in activation score in the *STAT3* ^*∆S*^ group (Mixed Linear Model treating region as a repeated measure, *p* = 0.037), with significant decrease of 65–90% in all six individual ipsilateral brain regions (*p* < 0.05 in *t*‐test).

### Astroglial deletion of pSTAT3 reduces brain damage following neonatal HI‐insult

**Figure 3:**
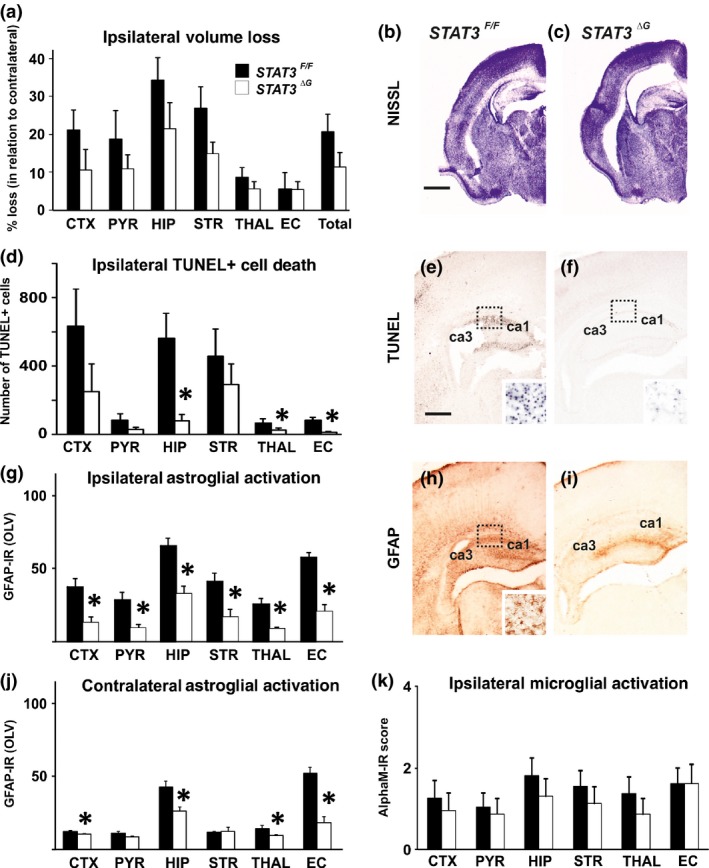
**Astroglial deletion of STAT3 with Cre recombinase under the control of GFAP promoter (*STAT3*^*ΔG*^) reduces cell death and reactive astrogliosis. All graph data are shown as Mean ± SEM. (a)** Ipsilateral brain tissue volume loss was not significantly affected despite the consistent lower average tissue loss values across the examined brain regions in the *STAT3* ^*∆G*^ animals. **(b and c)** Ipsilateral forebrain Nissl staining (Cresyl‐Violet, at rostral parietal level) of *STAT3* ^*F/F*^ (b) and *STAT3* ^*ΔG*^ (c). **(d)** Reduction in the number of TUNEL+ dying cells (per 20× eye‐field) at 48 h following HI, with significant, individual decrease (*t*‐test) in the *STAT3* ^*ΔG*^ group (*n *=* *12) compared to *STAT3* ^*F/F*^ littermate controls (*n *=* *15), in ipsilateral hippocampus (*p *=* *0.041), thalamus (*p* = 0.049) and external capsule (*p* = 0.040). Mixed Linear Model treating region as a repeated measure revealed *p* = 0.040. **(e and f)** Histochemical overview of TUNEL+ cell death of the ipsilateral forebrain in *STAT3* ^*F/F*^ (e) and *STAT3* ^*ΔG*^ (f) animals. Note the high density of typical pyknotic nuclear morphology of the TUNEL+ cells observed in the *STAT3* ^*F/F*^ group (e‐insert, hippocampus) and the reduced number of such cells in the *STAT3* ^*ΔG*^ group (f). **(g)** Strong reduction in ipsilateral reactive astrogliosis (GFAP immunoreactivity in OLV), with significant, individual decrease (*t*‐test) across all 6 ipsilateral forebrain regions – isocortex (*p *=* *0.0006), pyriform cortex (*p *=* *0.003), hippocampus (*p *=* *0.0002), striatum (*p *=* *0.003), thalamus (*p *=* *0.0003) and external capsule (*p *=* *1 × 10^−5^). Mixed Linear Model, treating region as a repeated measure revealed *p* = 0.001. **(h and i)** Low magnification ipsilateral overview in *STAT3* ^*F/F*^ (h) and *STAT3* ^*ΔG*^ (i) animals. The insert in H shows higher magnification of the dotted region in rostro‐parietal isocortex. Note the reduced levels of GFAP immunoreactivity in the *STAT3* ^*ΔG*^ animals. **(j)** Strong reduction in contralateral reactive astrogliosis with significant, individual decrease (*t*‐test) in contralateral isocortex (*p *=* *0.010), hippocampus (*p *=* *0.003), thalamus (*p *=* *0.040) and external capsule (*p* = 6 × 10^−6^). Mixed Linear Model, treating region as a repeated measure revealed *p* = 0.004. **(k)** Ipsilateral microglial activation score based on the αM integrin immunoreactivity was decreased in all examined regions but overall was not significantly affected. *STAT3* ^*F/F*^ (*n *=* *15) and *STAT3* ^*ΔG*^ (*n *=* *12) in all assessments. *p<0.05 *Scale bars*: e, f, h and i = 600 μm; inserts=30 μm; b and c = 1200 μm.

Compared with *STAT3* ^*F/F*^ control animals, deletion of both STAT3 gene copies with cre recombinase driven by astroglia‐specific GFAP promoter (*STAT3* ^*∆G*^) significantly reduced the extent of TUNEL+ cell death and astrogliosis. The effect on TUNEL+ cells is shown in Fig. 3a (Mixed Linear Model, treating region as a repeated measure, *p* = 0.02), with individual significant decrease of 30–87% in hippocampus, thalamus and external capsule (*p* < 0.05 in *t*‐test). However, despite the apparently consistent lower average tissue loss values across the examined brain regions (Fig. 3a, b and c) in *STAT3* ^*∆G*^ animals, astroglial deletion of STAT3 did not have a significant effect on ipsilateral brain tissue loss (Mixed Linear Model, treating region as a repeated measure, *p* = 0.217). Likewise, microglial activation based on the αM integrin immunoreactivity (Fig. 3k) had a trend towards reduced levels in all the examined regions but overall was not significantly affected (*p* = 0.229).

In contrast, astroglial pSTAT3 deletion strongly reduced HI‐induced up‐regulation of GFAP‐immunoreactivity in both ipsi‐ and contralateral brain regions (Fig. 3g,h, and i and Fig. 3j, respectively). The extent of this reduction was beyond that observed with *STAT3* ^*∆S*^ (Fig. 2k and l). Analysis with Mixed Linear Model, treating region as a repeated measure revealed *p* = 0.0001 on the ipsilateral and 0.0042 on the contralateral side, with individual significant decrease of 50–70% in all of the six regions on the occluded and 16–65% in four of the regions on the contralateral, non‐occluded side (*p* < 0.05 in *t*‐test).

### Inhibition of STAT3 Y705‐phosphorylation reduces HI‐mediated glial activation

**Figure 4:**
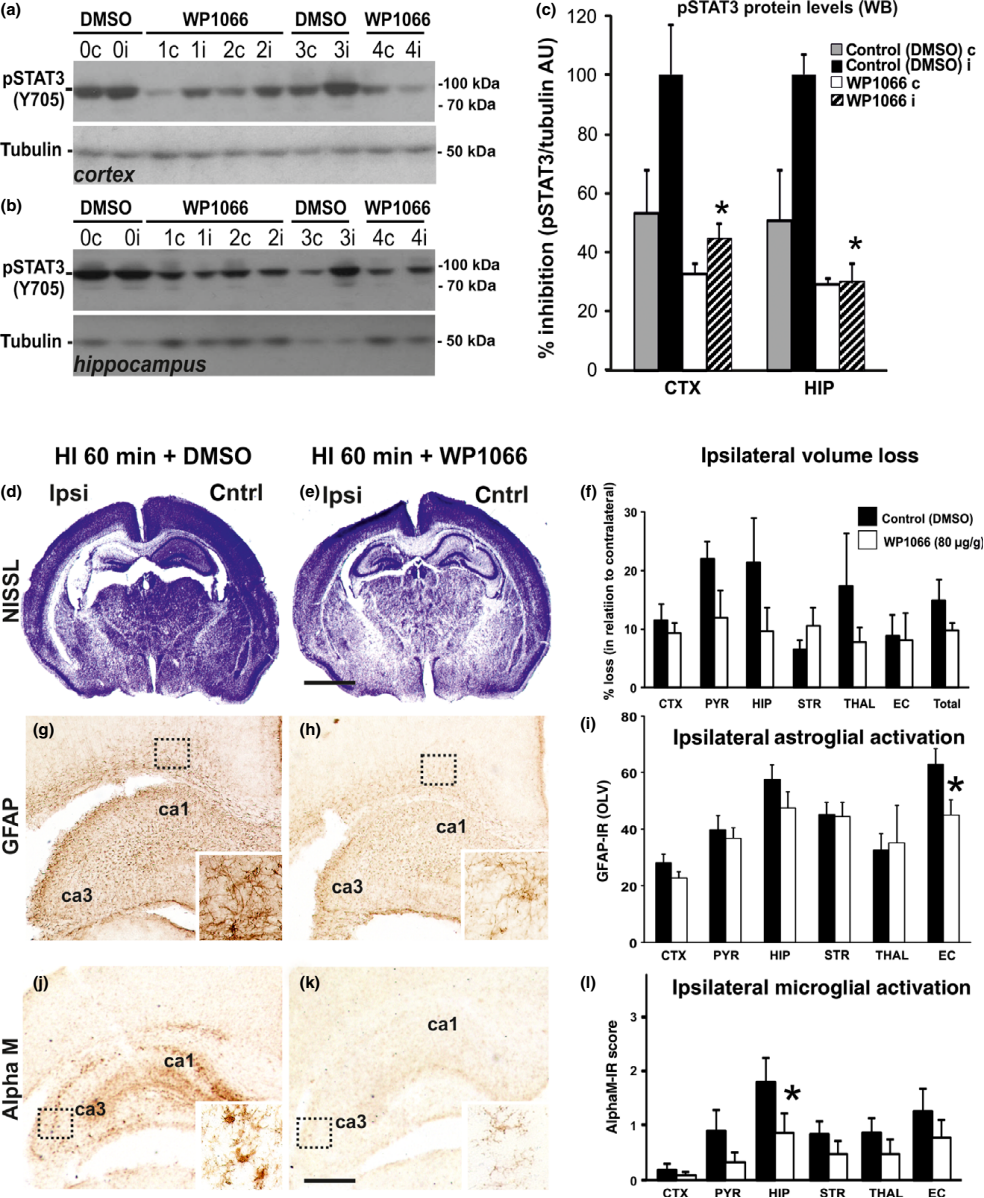
**Jak2‐inhibition with WP1066 reduces STAT3–phosphorylation (Y705), reactive astrogliosis and microglial activation following neonatal HI. (a–c)** Western Blots (a and b) of pSTAT3 (Y705) immunoreactivity in cerebral cortex (a) and hippocampus (b) and their densitometric quantification (c) in arbitrary units (AU) against a tubulin control (Mean ± SEM) at 1 h after 60 min HI‐insult. Intraperitoneal WP1066 injection at a combined dose of 80 μg/g BW (20 min prior and directly after 60 min HI‐insult), reduces pSTAT3 (Y705) levels in both cortex and hippocampus in the ipsi‐ (i) and contralateral (c) hemisphere (*n* = 6), when compared to the vehicle (DMSO) ‐injected littermate controls (*n* = 5). *Significant effect for WP1066 versus DMSO (unpaired *t*‐test) for ipsilateral cortex (*p *=* *0.005) and ipsilateral hippocampus (*p* = 0.005). **(d, e, g, h, j and k)** Nissl staining of whole forebrain (d and e), and ipsilateral immunoreactivity for astroglial GFAP (g and h) and αM+ microglia at rostro‐parietal level (j and k) 48 h after HI‐insult in animals injected with vehicle (DMSO) (d, g and j) or WP1066 (e,h and k). The inserts in (g and h) show WP1066‐induced reduction in GFAP immunoreactivity in external capsule and in (j and k) the disappearance of αM+ microglial phagocytes in the hippocampal CA3 subregion. **(f, i, and k)** Quantitative assessment of WP1066 effect vs DMSO (*n *=* *9 per group) on ipsilateral brain tissue volume loss (f), GFAP immunoreactivity in OLV (i) and αM microglial activation score (l). All graph data are shown as Mean ± SEM. In the Mixed Linear Model, treating region as a repeated measure did not reveal significant effect for volume loss (f), but demonstrated reduced astrogliosis (i, *p* = 0.019) and microglial activation score (l, *p* = 0.049) with significant, individual decrease (*t*‐test) in external capsule (GFAP‐immunoreactivity, i, *p* = 0.040) and in hippocampus (microglia, l, *p* = 0.048). *Scale bars*: d and e = 1200 μm, g, h, j and k = 300 μm; inserts = 30 μm.

Due to the early up‐regulation of pSTAT3 post‐HI (Fig. 1), the JAK2 inhibitor WP1066 was injected intraperitoneally just before and immediately after 60 min hypoxia to prevent STAT3 activation during the latent period. Compared to vehicle (DMSO)‐treated control animals, intraperitoneal application of WP1066 (80 μg/g BW) significantly reduced the protein levels of pSTAT3 (Y705) at 1 h post‐HI in both cortex (Fig. 4a and c) and hippocampus (Fig. 4b and c) on the ipsilateral side.

Pre‐ and post‐insult treatment with 80 μg/g WP1066 did not have significant effect on ipsilateral brain tissue loss at 48 h post‐HI in Mixed Linear Model, treating region as a repeated measure (*p *=* *0.257) (Fig. 4d–f). There was also no significant effect of WP1066‐treatment on the number of TUNEL+ cells (data not shown). However, reactive astrogliosis and microglial activation on the occluded side were affected. In the case of astroglial GFAP immunoreactivity (Fig. 4g–i), analysis with Mixed Linear Model, treating region as a repeated measure revealed *p* = 0.047, with individually significant decrease of 28% in external capsule (*p *<* *0.05 in *t*‐test). Microglial activation (Fig. 4j–l) based on αM integrin immunoreactivity was significantly reduced in animals treated with WP1066 (Mixed Linear Model, treating region as a repeated measure, *p* = 0.041), with individually significant decrease of 51% in ipsilateral hippocampus (*p* < 0.05 in *t*‐test). The insert in Fig. 4d shows the typical phenotype of strongly αM immunoreactive, phagocytic microglia in the vehicle (DMSO) control group, compared to ramified microglia with reduced αM immunoreactivity in the WP1066‐treated animal (Fig. 4e‐insert).

## Discussion

As shown in the current study, in a Rice‐Vannucci model of severe HI insult in postnatal (P7) mice, neuronal deletion of STAT3 (*STAT3* ^*ΔS*^ *)* clearly reduced forebrain cell death and tissue loss, as well as microglial and astroglial activation. Astroglia‐specific STAT3 deletion (*STAT3* ^*ΔG*^) blocked reactive astrogliosis and attenuated cell death, but had a more moderate effect on tissue loss and active microgliosis. A combined pre‐insult and immediate post‐insult blockade of JAK2 with systemically applied WP1066 inhibited the HI‐induced forebrain STAT3(705)‐phosphorylation and reduced HI‐brain damage based on some evidence for astroglial and microglial post‐HI response. However, this effect was weak and did not lead to a statistically significant reduction in brain tissue loss. Overall, our data suggest a critical role for STAT3, and possibly also a contribution in neonatal HI‐brain damage via Tyr705 phosphorylation.

There are significant differences in the response of different mouse strains to the Rice‐Vannucci model of neonatal HI (Sheldon *et al*. 1998; Rocha‐Ferreira *et al*. 2015), with C57/Bl6 demonstrating an increasing degree of injury with increasing duration of hypoxia, and SVJ129 demonstrating relative resistance to injury. As both the cell‐specific deletion strains used in our experiments were on mixed C57/Bl6 background with either FVB (astroglial) or SVJ129 (neuronal), and because of the reported susceptibility of the C57/Bl6 strain to the neonatal HI model, we chose to use the latter for the STAT3 inhibitor experiments.

The increase of pSTAT3 (Y705) in the current study, was specific for HI, peaking at 1–4 h following insult (Fig. 1e and g). We did not observe STAT3 activation in the brains of naïve, animals subjected to hypoxia alone or of those with unilateral carotid occlusion alone (Fig. 1a and b), suggesting a HI‐dependent pattern of STAT3 activation (Shrivastava *et al*. 2013). Interestingly, the early pSTAT3 presence at 1 h following HI was specific for neurons but not for the GFAP+ astrocytes (Fig. 1j and n) or the αM+ microglia (Fig. 1k and o), preceding the previously observed up‐regulation from 3 to 72 h post‐HI in micro‐ and astroglial populations (Shrivastava *et al*. 2013), and suggesting an additional, time‐dependent and cell‐specific pattern of STAT3 activation. The pSTAT3‐localization around neuronal cell body‐near processes (Fig. 1h and i and l and m) could be explained with a previously described role for this retrogradely transported axonal molecule in injury‐induced axonogenesis of hippocampal neurons (Ohara *et al*. 2011).

Total STAT3 protein levels remained unaffected and similar to the expression observed in naïve animals (data not shown), implying that the functional effect of STAT3 is probably elicited by some post‐translational modification and/or translocation, cytoplasmic translation and nuclear transcription (Nicolas *et al*. 2012; Haghikia *et al*. 2014). Most of the immediate/early HI damage in the Rice‐Vannucci model occurs on the occluded side (obvious from Nissl histology in Fig. 2c and d or phagocytic, aX+ microglia in Fig. 2m–p). However, pSTAT3‐activation is bilateral (Figs 1a and b and 2a and b) with contralateral intensity possibly depending on the strain background. This bilateral activation suggests that STAT3 is not a directly pathogenic effector molecule but rather exerts a permissive, but nevertheless substantial, predisposing role in mediating HI‐injury. Although the contralateral side in the Rice‐Vannucci model is often used as intra‐animal control reference for ipsilateral damage (Vannucci *et al*. 1988; Towfighi *et al*. 1994; Skoff *et al*. 2007; Shrivastava *et al*. 2012), some studies report bilateral change in the expression of some cytokines, for example Hypoxia Inducing Factor alpha and P‐Akt (Shrivastava *et al*. 2012), suggesting that hypoxia can regulate some mediators contributing, but not sufficient to cause long‐term damage. The same study reports some degree of delayed contralateral atrophy in hippocampus and corpus callosum (Shrivastava *et al*. 2012). Severe HI‐insult in the Rice‐Vannucci model following unilateral carotid occlusion and prolonged (60 +  min) hypoxia is associated not only with substantial acidosis in the territory of the ipsilateral medial cerebral artery but also with very pronounced contralateral pH drop (Kendall *et al*. 2011b), which could contribute to the rapid STAT3 activation on the non‐occluded side observed in the current study. Moreover, the elevated, contralateral levels of pSTAT3, and the fact that neuronal as well as astroglial STAT3 deletion inhibit contralateral astrogliosis (Figs 2j and 3d) and microgliosis (Fig. 2n) also points to a more long‐term involvement in pathways underlying delayed contralateral atrophy (Shrivastava *et al*. 2012).

We used 3 strategies to interfere with brain STAT3 function, i.e. removal of STAT3 gene expression in neurons (*STAT3* ^*ΔS*^), in GFAP+ astroglia (*STAT3* ^*ΔG*^) and blocking JAK2‐mediated Y705‐phosphorylation with WP1066. The pharmacological inhibition produced a relatively moderate effect. Compared to vehicle (DMSO)‐treated controls, the combined pre‐HI and immediate post‐HI intraperitoneal injection of WP1066 significantly reduced the levels of pSTAT3 in cortex and hippocampus (Fig. 4a–c), demonstrating that WP1066 does penetrate the blood brain barrier at a physiologically active dose (Hussain *et al*. 2007). It is possible that this reduction is due to an incomplete, approximately 70% decrease in Y705‐phosphorylation thus permitting some residual activity, compared to complete removal in neurons or astrocytes expressing cre recombinase. Moreover, there are several different ways involved in STAT3 activation – phosphorylation at Y705 and S727, dimerization, nuclear translocation, etc. (Haghikia *et al*. 2014) – and interference with just one could produce a moderate result. Interestingly, cell‐specific removal of the floxed STAT3 gene, as well as pharmacological inhibition result in partial overlap of the histopathology phenotypes, but there are pronounced differences in the magnitude of their impact suggesting either direct or secondary effects.

Removal of STAT3 in astrocytes resulted in a consistent, general decrease in reactive astrogliosis across different regions of the ipsilateral and contralateral hemisphere, underscoring the essential role of this transcription factor in astroglial activation and induction of GFAP (Fukuda *et al*. 2007; Herrmann *et al*. 2008; Hong and Song 2014). Previous studies have suggested that reactive astrogliosis is neuroprotective and enhances repair. Astroglial STAT3 deletion interferes with synaptic plasticity on regenerating facial motor neurons via astrocyte TSP1 (Tyzack *et al*. 2014), delays oligodendroglial maturation in endotoxin‐induced neonatal white matter damage (Nobuta *et al*. 2012), and increases inflammation and lesion volume, with attenuated motor recovery in spinal cord injury (Herrmann *et al*. 2008). However, in the current model, deletion of astroglial STAT3 resulting in suppression of astrogliosis was associated with clearly reduced TUNEL+ cell death in the ipsilateral hippocampus, thalamus and external capsule (Fig. 3d,e and f). Although the effects were less prominent than with neuron‐specific deletion (Fig. 2e, g and h), and the decrease in tissue loss and microglial activation did not reach the statistical significance needed for repeated measures, in our study reduced astrogliosis in HI was associated with decrease in cell death and did not increase inflammation or tissue loss, thus contradicting the effects observed in other experimental brain pathologies (Herrmann *et al*. 2008; Nobuta *et al*. 2012).

In contrast to GFAP promoter‐driven deletion, pSTAT3‐deletion via the neuronal synapsin promoter produced a general decrease of cell death across all examined regions of the ipsilateral hemisphere, and significantly reduced tissue loss in cerebral isocortex and striatum, and overall ipsilateral forebrain volume loss. It also resulted in a significant reduction in microglial αM activation score and the number of αX+ phagocytes. Microglia do not express synapsin during embryonic or post‐natal development (Kügler *et al*. 2001). The neuronal effects on reactive astrogliosis are fairly moderate (Fig. 2i and j), and the decrease in microgliosis in *STAT3* ^*ΔG*^ animals did not reach statistical significance (Fig. 3k). Therefore, the observed reduction in microglial activation in the *STAT3* ^*ΔS*^ animals is probably a consequence of reduced neuronal signalling and/or the strongly decreased cell death and tissue loss. Following neonatal HI‐injury activated microglia, similarly to other pathologies, are a major marker of damage (Ohno *et al*. 1995) and source of inflammatory cytokines contributing to cell death, tissue loss and astroglial activation, as well as an important contributor to endogenous defence mechanisms through effective elimination of apoptotic neurons (Faustino *et al*. 2011). Activated microglial cells produce interleukin‐6 (IL6) (Lambertsen *et al*. 2012), a strong astroglial activator *in vitro* (Benveniste *et al*. 1995) and *in vivo* (Campbell *et al*. 1993; Galiano *et al*. 2001). Moreover, elevated levels of microglial IL6 following neonatal HI‐injury correlate with increase in pSTAT3 +  astrocytes (Shrivastava *et al*. 2013). Interestingly, WP1066 strongly inhibits microglial IL6 production (Lambertsen *et al*. 2012), which in the current study could contribute to the WP1066‐dependent reduction in reactive astrogliosis.

Overall, neuronal and astroglial STAT3 are clearly involved in the pathways underlying cell death, tissue loss and gliosis following neonatal HI but they do differ with respect to the target of their effect. Y705‐phosphorylation does contribute to HI histopathology; however a better understanding of this and the other pathway(s) through which STAT3 activation is involved in HI‐brain damage would considerably improve the therapeutic potential of interfering with STAT3 in a clinical setting.
